# Gene expression profiling of whole blood in ipilimumab-treated patients for identification of potential biomarkers of immune-related gastrointestinal adverse events

**DOI:** 10.1186/1479-5876-11-75

**Published:** 2013-03-22

**Authors:** Vafa Shahabi, David Berman, Scott D Chasalow, Lisu Wang, Zenta Tsuchihashi, Beihong Hu, Lisa Panting, Maria Jure-Kunkel, Rui-Ru Ji

**Affiliations:** 1 Bristol-Myers Squibb Company, Princeton, NJ, USA

**Keywords:** Metastatic melanoma, Ipilimumab, Gastrointestinal, Colitis, Adverse events, Gene expression profiling, Biomarkers, Gene expression, GI irAE, Diarrhea

## Abstract

**Background:**

Treatment with ipilimumab, a fully human anti-CTLA-4 antibody approved for the treatment of advanced melanoma, is associated with some immune-related adverse events (irAEs) such as colitis (gastrointestinal irAE, or GI irAE) and skin rash, which are managed by treatment guidelines. Nevertheless, predictive biomarkers that can help identify patients more likely to develop these irAEs could enhance the management of these toxicities.

**Methods:**

To identify candidate predictive biomarkers associated with GI irAEs, gene expression profiling was performed on whole blood samples from 162 advanced melanoma patients at baseline, 3 and 11 weeks after the start of ipilimumab treatment in two phase II clinical trials (CA184004 and CA184007). Overall, 49 patients developed Grade 2 or higher (grade 2+) GI irAEs during the course of treatment. A repeated measures analysis of variance (ANOVA) was used to evaluate the differences in mean expression levels between the GI irAE and No-GI irAE groups of patients at the three time points.

**Results:**

In baseline samples, 27 probe sets showed differential mean expression (≥ 1.5 fold, *P* ≤ 0.05) between the GI irAE and No-GI irAE groups. Most of these probe sets belonged to three functional categories: immune system, cell cycle, and intracellular trafficking. Changes in gene expression over time were also characterized. In the GI irAE group, 58 and 247 probe sets had a ≥ 1.5 fold change in expression from baseline to 3 and 11 weeks after first ipilimumab dose, respectively. In particular, on-treatment expression increases of CD177 and CEACAM1, two neutrophil-activation markers, were closely associated with GI irAEs, suggesting a possible role of neutrophils in ipilimumab-associated GI irAEs. In addition, the expression of several immunoglobulin genes increased over time, with greater increases in patients with grade 2+ GI irAEs.

**Conclusions:**

Gene expression profiling of peripheral blood, sampled before or early in the course of treatment with ipilimumab, resulted in the identification of a set of potential biomarkers that were associated with occurrence of GI irAEs. However, because of the low sensitivity of these biomarkers, they cannot be used alone to predict which patients will develop GI irAEs. Further investigation of these biomarkers in a larger patient cohort is warranted.

## Background

Ipilimumab, a fully human monoclonal antibody that blocks cytotoxic T-lymphocyte antigen-4 (CTLA-4) [1], has been approved by the U.S. Food and Drug Administration (FDA) and several other regulatory agencies for the treatment of advanced metastatic melanoma (MM). The efficacy of ipilimumab has been demonstrated in a number of phase II and two phase III clinical trials in MM patients, where a significant prolongation of overall survival has been reported [2,3].

Treatment with ipilimumab is associated with a spectrum of AEs which are immune mediated and called immune-related adverse events (irAEs). Gastrointestinal (GI) irAEs such as diarrhea and colitis are among the most common ipilimumab-associated irAEs [4]. In most cases the onset of GI irAEs occurs after the second or third dose of ipilimumab [5] and these irAEs are managed according to established treatment guidelines. In a previous report, examination of colon biopsies in a safety-focused clinical trial (CA184007) revealed abundant focal neutrophilic cryptitis and neutrophilic infiltration in the lamina propria of affected tissues from patients experiencing GI irAEs. Although administration of high doses of steroids often leads to successful and safe management of the majority of these irAEs [5-7], identification of biomarkers that may predict (before or soon after the start of the treatment) these irAEs might improve patient care. In this context, peripheral blood biomarkers would be preferred, since collection of peripheral blood is less invasive than a colonic biopsy.

To understand the underlying causes of ipilimumab-associated GI irAEs and identify potential predictive biomarkers, gene expression profiling was performed on whole blood samples collected from metastatic melanoma patients before and during ipilimumab treatment in two phase II clinical trials, CA184004 and CA184007 [6,8]. A number of cell cycle- and immune-related genes were found to have higher expression at baseline and post-baseline in those patients who experienced GI irAEs. In particular, increases in expression of neutrophil activation markers, CD177 and CEACAM1, were found to be associated with the occurrence of GI irAEs. In addition, greater increases in the expression of immunoglobulin-related genes were detected at weeks 3 and 11 in patients with GI irAEs than in those without. These results are consistent with our understanding of the mechanisms underlying ipilimumab-associated GI irAEs and provide a list of potential peripheral blood biomarkers for early prediction of these irAEs.

## Methods

### Study design

The multicenter, phase II clinical trial CA184004 enrolled 82 previously-treated or untreated patients with stage III (unresectable) or IV melanoma, randomized 1:1 into 2 arms to receive up to 4 intravenous infusions of either 3 or 10 mg/kg ipilimumab every 3 weeks in an induction phase. In trial CA184007, treatment-naïve or previously treated patients with stage III (unresectable) or IV melanoma (N = 115) received open-label ipilimumab (10 mg/kg every 3 weeks for four doses) and were randomized to receive concomitant blinded prophylactic oral budesonide (9 mg/d with gradual taper through week 16) or placebo (4). Exclusion criteria included the use of any immuno-suppressing treatments including corticosteroids (patients on stable doses of hormone replacement therapy were exempt), cyclosporine, mycophenolate mofetil (CellCept®), as well as chemotherapy and radiation, within 4 weeks prior to the first ipilimumab dose. Complete study design, patient characteristics and endpoint reports of these trials were described elsewhere [6,8]. Gene expression profiles from twenty patients in CA184078 [9] who were treated with ipilimumab monotherapy at 10 mg/ml were used as a confirmation data set for the present analysis. These studies were conducted in accordance with the ethical principles originating from the current Declaration of Helsinki and consistent with International Conference on Harmonization Good Clinical Practice and the ethical principles underlying European Union Directive 2001/20/EC and the United States Code of Federal Regulations, Title 21, Part 50 (21CFR50). The protocols and patient informed consent forms received appropriate approval by all Institutional Review Boards or Independent Ethics Committees prior to study initiation. All participating patients (or their legally acceptable representatives) gave written informed consent for these biomarker-focused studies.

### Adverse event and clinical activity evaluation

Safety was evaluated using the National Cancer Institute Common Terminology Criteria for Adverse Events, based on adverse events (AEs), physical examinations, and clinical laboratory assessments. Drug-related gastrointestinal AEs consistent with immune-mediated events and the intrinsic biological activity of ipilimumab were examined and reported. Adverse events were recorded based on MedDRA v10.0 system organ class and Preferred Terms. Clinical activity (CA) was defined as confirmed complete response, confirmed partial response, or stable disease ending not earlier than 24 weeks from date of first ipilimumab dose. A complete description of irAE and CA evaluations for these trials has been reported elsewhere [6,8].

### Absolute peripheral blood neutrophil count (APBNC)

Neutrophils were quantified as a component of the standard hematology panel. Absolute neutrophil counts were available at various time points for most patients.

### Affymetrix gene expression profiling

Whole blood samples for gene expression profiling were collected just prior to first dose of ipilimumab (baseline), and 3 and 11 weeks after first ipilimumab dose. Total RNA was extracted from whole blood samples using the PAXgene Blood RNA MDX kit with a BioRobot Universal System (Qiagen, Valencia, CA), and purified by RNeasy MinElute Cleanup kit using QIAcube (Qiagen, Valencia, CA). RNA concentration was measured by NanoDrop spectrophotometer and RNA integrity was evaluated on the Agilent 2100 Bioanalyzer (Agilent Technologies, Santa Clara, CA). Complementary DNA preparation and hybridization on HT-HG-U133A 96-array plates followed manufacturer’s protocols (Affymetrix, Santa Clara, CA). Affymetrix raw data (.CEL files) were normalized with the robust multi-array analysis (RMA) algorithm, obtained from http://www.bioconductor.org, version 1.20.2. Appropriate Affymetrix control probe sets were examined to ensure quality control for the cDNA synthesis and hybridization steps. Principal component analysis (PCA) was subsequently performed to detect outlier samples (single samples that account for a high degree of variation in the data). No sample was removed as an outlier. Anti-log RMA values were used in subsequent statistical analyses. For the combined data from studies CA184004 and CA184007, 12518 of the 22215 non-control probe sets had maximum expression level (RMA normalized) of less than 32. These probe sets, with low expression levels across all samples, were excluded from further analysis.

### Gene expression data analyses

GI irAE status was available for all 197 treated patients. Gene expression data were available for 188 of these patients. For 162 of the patients, expression data were available for at least 2 of the 3 time points. Only these patients were included in subsequent analyses. Of these patients, 113 were classified in a No-GI irAE group, which included patients with a worst-grade GI irAE of 0 or 1. A total of 49 patients with grade 2 or greater (grade 2+) GI irAEs were classified in a GI irAE group. For the No-GI irAE and GI irAE groups, respectively, baseline expression data were available for 108 and 49 patients, week 3 data were available for 108 and 47 patients, and week 11 data were available for 78 and 30 patients.

A repeated measures analysis of variance (ANOVA) model was fit in Partek Genomics Suite 6.6 (http://www.partek.com), with anti-log normalized expression level as dependent variable. Explanatory variables included patient, time point within patient as a 3-level factor, and binary GI irAE status, with no time-by-status interaction. Because GI irAEs were observed in all treatment arms, and because of the relatively small sample sizes in individual trials, data from patients in the two trials were combined to increase statistical power to detect associations. Statistical inference based on this model focused on two hypothesis tests: a test of the null hypothesis that mean gene expression (averaged over time) was the same in the two GI irAE status groups, and a test of the null hypothesis that mean gene expression (averaged over GI irAE status) was the same for the three time points. An uncorrected *P* value of 0.05 was used as a cutoff to select probe sets with mean expression differences between comparison groups. The qvalue package (v1.20.0) in the R statistical computing environment (v2.15.0, http://www.r-project.org) was used to estimate false discovery rates (FDRs). Expression of selected genes was confirmed by quantitative polymerase chain reaction (qPCR) as described previously [10] using pre-designed probes.

### Gene pathway analysis

Functional interpretation of differentially expressed genes was computed using Ingenuity Pathway Analysis (IPA) software (Ingenuity Systems), as described previously [10].

## Results

### Gene expression profile of pre-treatment samples

Expression of each of 9697 non-control probe sets was analyzed individually. Genes associated with GI irAE status (grade 2+ vs. not) were selected by assessing the difference in mean pre-treatment expression between the GI and No-GI irAE groups. Two selection criteria were applied: a *P* value ≤ 0.05 for the hypothesis test comparing the GI and No-GI irAE groups, and a minimum mean pre-treatment expression ratio of 1.5. For these tests, the *P* value threshold of 0.05 corresponded to an estimated FDR of 0.50. A set of 27 probe sets representing 24 unique genes met these criteria (Additional file 1: Table 1A). This list included a number of immune-related genes, such as CD3E, IL2RG [11], CD37 [12], CD4, IL32 [13], and RAC2 [14]; cell cycle- and proliferation-associated genes such as SPTAN1 [15], BANF1 [16], BAT1 [17], PCGF1 [18], FP36L2 [19], and WDR1 [20]; and genes involved in intracellular vesicle trafficking such as PICALM [21], SNAP23 [21], and VAMP3 [22]. Some of these molecules such, as IL32, SNAP23 and RAC2, have been reported either to be present in neutrophils or to regulate their function [13,14,21].

### Gene expression profiles of post-baseline samples

**Figure 1:**
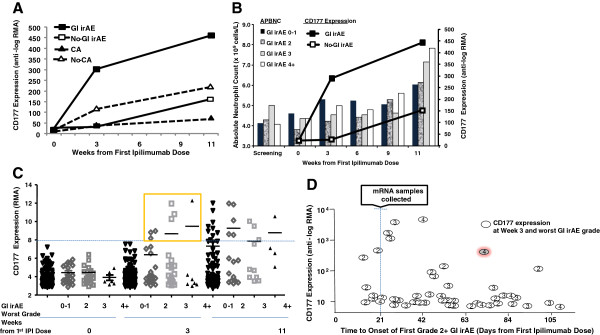
**CD177 expression during ipilimumab treatment. **(**A**) Mean expression of CD177 during ipilimumab treatment in patients who experienced grade 2+ gastrointestinal immune-related adverse events (GI irAE), or did not (No-GI irAE). Mean gene expression in patients showing clinical activity (CA) or not (No-CA) also are plotted; (**B**) Mean expression of CD177 during ipilimumab treatment in patients who experienced grade 2+ GI irAE, or did not (No-GI irAE), and mean absolute peripheral blood neutrophil counts (APBNC) during the same time period, by worst-grade GI irAE (bars); (**C**) CD177 expression level (RMA-normalized value) versus worst-grade GI irAE, by sample-collection time; (**D**) CD177 expression level (anti-log RMA-normalized value) at week 3 versus time to onset of first grade 2+ GI irAE. Numbers in the circles describe the worst grade of GI irAE reported during the course of treatment. Highlighted circle represents CD177 expression in the patient who experienced a fatal grade-4 GI irAE.

To identify early on-treatment predictors of ipilimumab-associated GI irAEs, post-baseline expression levels of the 9697 non-control probe sets were compared between the GI irAE and No-GI irAE groups. Thirty five and 47 probe sets were identified to have a mean expression ratio of at least 1.5 for the week 3 and 11 samples, respectively, and a *P* value ≤ 0.05 (FDR = 0.50) for the hypothesis test comparing the GI and No-GI irAE groups. Since most ipilimumab-associated GI irAEs occur after the second or third dose of ipilimumab, the 35 probe sets differentially expressed at week 3 are of particular interest, as they might serve as early predictors to help identify patients who experience GI irAEs after the second dose, given at week 3 (Additional file 1: Table 1B). The probe set that exhibited the largest differential expression corresponded to the neutrophil-specific marker, CD177, a glycosyl-phosphatidylinositol (GPI)-linked cell surface molecule [23]. There was no difference in baseline expression of CD177 between patients in the GI and No-GI irAE groups. However, significantly higher CD177 expression was found after only one dose of ipilimumab in the GI irAE group [12.2 fold higher in the GI irAE group than in the No-GI irAE group at week 3 (*P* = 7.6E-03)]. In addition, increase in mean expression of CD177 from baseline to week 3 was much greater in the GI irAE group than in the No-GI irAE group (Additional file 1: Table 2). The mean increase in CD177 expression was not associated with clinical activity (CA) of ipilimumab (*P* = 0.23) (Figure 1A). Expression levels of CD177 in these samples were confirmed by quantitative PCR, showing statistically significant differences between the GI irAE and No-GI irAE groups (unadjusted *P* < 0.005) or changes over time (unadjusted *P* < 0.0001). These data suggest that CD177 was not only a potential early predictor of GI irAEs but that increase in CD177 gene expression might also be a consequence of treatment with ipilimumab independent of its clinical activity.

### Changes in CD177 expression in relation to peripheral blood absolute neutrophil count (PBANC)

Since CD177 is a neutrophil surface marker, we examined the relationship between PBANC and expression levels of CD177. Mean PBANC increased gradually with ipilimumab treatment, with the largest apparent increases occurring between weeks 9 and 11 in patients with higher-grade GI irAE (Figure 1B, bars). The increase in mean expression of CD177 preceded any significant increase in PBANC (Figure 1B, lines), suggesting that CD177 expression might be a more sensitive early predictor of GI irAEs associated with ipilimumab treatment than PBANC.

### Evaluation of CD177 as an early predictor of GI irAEs

Expression of CD177 at week 3 had large inter-individual variability, with considerably higher values in 7 patients than in the rest (Figure 1C). All seven patients with high levels of CD177 expression (RMA-normalized expression level ≥ 8) at week 3 had grade 2+ GI irAEs during the course of treatment, suggesting high specificity of this biomarker above this threshold. However, many patients with grade 2+ GI irAEs had a normalized expression level < 8 for CD177 at week 3, suggesting low sensitivity of the biomarker in predicting GI irAEs (Additional file 2: Figure S1). In week 11 post-baseline samples, high expression levels of CD177 were found in both the GI irAE and No-GI irAE groups. Many patients with grade 2+ GI irAEs had already discontinued treatment before this time point, so the data from week 11 might be biased by informative drop-out.

We also explored the timing of the onset of GI irAEs in individual patients to establish the value of CD177 expression as an early predictor. Out of 44 patients with grade 2+ GI irAEs who had matching gene expression data at week 3, 6 patients reported the first GI irAE before or on day 21 ± 3 days (the nominal date of blood sample collection), whereas the other 38 patients had GI irAEs reported after this date (Figure 1D). The four highest values of CD177 expression were detected in patients who reported the first grade 2+ GI irAE between days 26 (2–5 days after blood sample collection) and 43 after receiving the first dose of ipilimumab. Of note, one of the patients who had high CD177 expression at week 3 reported a grade 2 GI irAE on day 72 (Figure 1D, marked in gray shade circle), but progressed to a grade 4 which ultimately led to fatal GI perforation. These data suggest that, although considerable increase in CD177 gene expression was closely associated with the onset of GI irAEs, early increases might also predict GI irAEs that could develop much later. However, since CD177 had low sensitivity, this biomarker could not identify most future GI irAEs.

### Association between expression of CD177 and other neutrophil-associated genes

**Figure 2:**
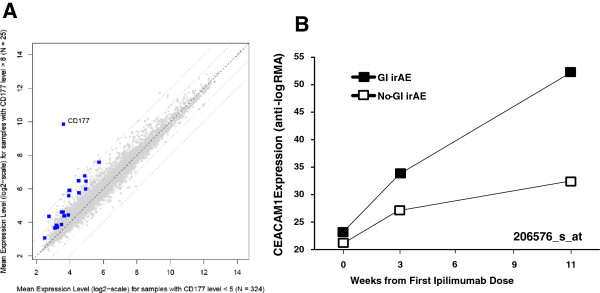
**Expression of granule-associated genes. **(**A**) Mean expression level for samples with high CD177 expression (> 256) versus that for samples with low CD177 expression (< 32), for each of the 9697 probe sets passing the low-expression filter. Each point represents one probe set. Blue squares highlight CD177 and 19 probe sets for 13 unique neutrophil granule-associated genes. Diagonal reference lines indicate mean expression ratios of 4, 2, 1, 0.5, and 0.25. Expression of genes with points above the central dashed line was positively associated with CD177 expression. (**B**) Mean gene expression (anti-log RMA-normalized value) of CEACAM1 (probe set: 206576_s_at) during ipilimumab treatment in patients with (GI irAE) or without (No-GI irAE) a grade 2 or greater GI irAE.

CD177 is a glycoprotein expressed by neutrophils, neutrophilic metamyelocytes, and myelocytes, but not by any other blood cells [24,25]. Therefore we specifically searched for other neutrophil-associated genes, to better understand the implication of this granulocyte subtype as an early predictor of GI irAEs. These included genes encoding for granule-associated proteins such as olfactomedin 4 (OLFM4) [26], azurocidin 1 (AZU1), lactoferrin (LTF) [27], cathelicidin (CAMP), myeloperoxidase (MPO) [28], bactericidal/permeability increasing protein (BPI), defensin (DEFA4) [29], neutrophil elastase (ELANE) [30], cathepsin G (CTSG) [30], CEACAM6, CEACAM8 [31], and CEACAM1 (which mediates neutrophil adhesion to endothelial cells and facilitates their transmigration into tissues) [32]. The GeneChip HT-HG-U133A includes probe sets for many of these genes Additional file 1: Table S3. Although an apparent greater expression of each of these genes was found in those samples with high levels of CD177 expression (Figure 2A), only the expression of CEACAM1 was significantly linearly correlated with that of CD177 at week 3 (r = 0.75, *P* = 7.2E-30 between 219669_at, probe set for CD177, and 206576_s_at, probe set for CEACAM1). Consequently, the pattern of mean gene expression of CEACAM1 over time was similar to that for CD177 (Figures 1A and 2B).

### Pathway analysis of changes in gene expression from baseline to week 11

**Figure 3:**
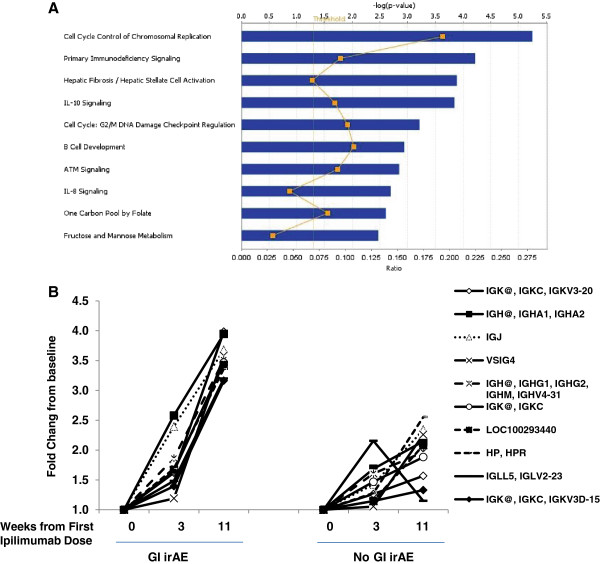
**Expression of immune-related genes during ipilimumab treatment. **(**A**) Functional interpretation of genes differentially expressed between baseline and week 11, as computed by Ingenuity Pathway Analysis (IPA) software (Ingenuity Systems). The *P* value reflects the significance of the enrichment of input genes in the functional category or pathway of interest. For every canonical pathway, IPA also provides the ratio of the number of genes from the input list that are annotated to the pathway to the total number of genes annotated to the pathway. (**B**) Fold change over time in expression of immunoglobulin-associated genes in the GI irAE and No-GI irAE groups. Genes shown were selected from the top 50 genes having the most prominent mean differences in on-treatment expression between the two GI irAE groups.

Fifty-eight and 247 probe sets were identified as having at least a 1.5-fold change from baseline in week 3 and 11 samples, respectively, and *P* ≤ 0.05 (FDR = 0.055) for the test of a time effect on expression (Additional file 1: Tables 2 and S1). We performed a pathway analysis using the IPA software on the 247 differentially expressed probe sets since the size of this gene set was amenable to such analysis. The top biological processes that exhibited changes during ipilimumab treatment included pathways of cell proliferation and metabolism, and immune-related pathways such as IL-10 signaling, IL-8 signaling, and B cell development Figure 3A).

### Increase in expression of immunoglobulin genes in post-baseline samples

While CD177 exhibited the largest change from baseline at both time points, immunoglobulin-related genes dominated both lists, including IGHA1, IGHA2, IGHG1, and IGHV4-31, all of which showed significant increases in expression 11 weeks after baseline. Slight increases in expression of some of these genes were already apparent in week 3 samples, suggesting an early onset of antibody response with much larger expansion at later time points (Additional file 1: Table 2 and Figure 3B). Notably, increases in expression of these genes over time were more prominent in the GI irAE groups than in the No-GI irAE group. There was no corresponding increase in B-cell marker genes (such as CD20), suggesting that the cell types responsible for the increased expression of immunoglobulin genes may not have been B-cells, but later-stage B-lineage cells such as plasma cells [33].

### Confirmation of findings in an independent study (CA184078)

Whole blood samples from the clinical study CA184078 were independently analyzed using the same statistical model. In this study, 20 patients were treated with ipilimumab monotherapy at 10 mg/kg every 3 weeks for 4 doses. The mean CD177 expression ratio comparing GI irAE and No-GI irAE groups at week 3 and 11 was 4.3 and 12.0, respectively, with no significant difference at baseline. In the GI irAE group, the mean fold change from baseline to week 3 and 11 was 4.8 and 15.3, respectively. By contrast, in the No-GI irAE group, these changes were negligible (1.1 and 1.2, respectively). Expression changes similar to those seen in the other two studies were observed for CEACAM1 and most of the granule-associated genes, with significant changes from baseline to week 11 in the GI irAE group but not in the No-GI irAE group (Additional file 1: Table S2).

## Discussion

Treatment with ipilimumab has been shown to prolong overall survival in advanced melanoma patients in 2 phase 3 trials [2,34]. Gastrointestinal irAEs such as diarrhea and colitis are among the most common and sometimes severe forms of adverse events associated with ipilimumab. These adverse events are currently managed following treatment guidelines. However, identification of predictive biomarkers is important as it may enhance the management of these toxicities and improve patient care. The present study was undertaken to identify such biomarkers in peripheral blood. In a previous report of the CA184007 trial in which colonic biopsies were collected, changes in the colonic mucosa following onset of diarrhea appeared more severe than those observed in the pre-specified biopsies following the first dose of ipilimumab. Histopathologic examination of the biopsies revealed active colitis with marked neutrophilic infiltration into lamina propria to be the most striking characteristic of the affected tissue. In these biopsies, foci of neutrophilic cryptitis, crypt abscesses, glandular destruction, and erosions of the mucosal surface were also apparent early after the start of treatment in those patients who presented with GI irAE symptoms [5]. However, collection of colonic biopsies is considered an invasive procedure and therefore, peripheral-blood surrogate markers would be preferred.

GI irAE onset is most commonly observed after the second or third doses of ipilimumab. Gene expression profiling using pre-treatment blood samples identified a few immune-related genes with higher baseline expression in patients who developed GI irAEs than in those who did not, including CD3E, IL2RG, CD4, CD37 and IL-32. Interleukin 32 (natural killer protein-4) might be the most interesting gene from this list, as this cytokine has been reported to play a central role in acute flares of inflammatory bowel disease [35], as well as other autoimmune diseases such as Rheumatoid arthritis [36]. IL32 is selectively expressed by activated cytotoxic T and natural killer (NK) cells [37], potentiates the effect of IL-2 and IL-18 and activates the innate immune system (monocytes and macrophages) to secrete chemotactic factors such as tumor necrosis factor-alpha (TNF-α), CXCL2 (11), and IL-8. A direct effect of IL-8 on the activation of neutrophils, the expression or activation of adhesion molecules in the neutrophil cell membrane, and the subsequent degranulation process has been reported in a number of studies [38,39]. In the current study, although treatment-induced changes in the expression of IL-8 were not significant, pathway analysis found IL-8 signaling to be one of the top 10 pathways changed during treatment (Figure 3A).

An increase in expression of neutrophil-associated genes was noticeable shortly after the start of treatment. In particular, significantly greater mean expression of the neutrophil surface marker CD177 was detected in a subset of patients who experienced GI irAEs of grade 2 or greater. Neutrophil granulocytes are the most abundant type of white blood cells in mammals and form an essential part of the innate immune system. During the acute phase of inflammation, particularly as a result of bacterial infection, environmental exposure [40], and some cancers [41,42], neutrophils are among the first-responders of inflammatory cells to migrate towards the site of inflammation. They transmigrate through the blood vessels, then through interstitial tissue, following chemical signals such as IL-8, C5a, and Leukotriene B4 in a process called chemotaxis [43]. CD177 is a unique marker for neutrophils [24,25] and is up-regulated upon neutrophil activation during acute inflammatory responses toward stimuli such as bacterial infections [44]. Significant increase in the mean expression of this marker was detected in patients who experienced moderate to severe GI irAEs, independently from their clinical response to ipilimumab. Indeed, the seven patients with the highest expression levels of CD177 (normalized expression level > 8) at week 3 were those who already had or would experience GI irAEs within a few days to three weeks after that time point. Currently, GI irAEs during ipilimumab treatment are managed, according to treatment guidelines, by cessation of ipilimumab in combination with treatment with corticosteroids or TNF-α blockade. CA184004 was one of the earlier ipilimumab monotherapy trials, where these guidelines were not yet fully in place. In that trial, two patients died of severe GI irAEs and intestinal perforation. One of these patients provided both baseline and week 3 blood samples for gene expression analysis. A 42.5-fold increase in CD177 expression from baseline to week 3 was apparent almost 50 days before the onset of the first GI irAE episode, suggesting the early predictive value of this biomarker for severe GI irAEs. CD177 has been shown to recognize an endothelial cell junction molecule, platelet endothelial cell adhesion molecule-1 (PECAM-1), which contributes to interactions between neutrophils and endothelial cells, mediating trans-endothelial migration in the context of inflammatory cell recruitment [45].

Another marker expressed by activated neutrophils, CEACAM1, also showed a consistent increase in mean expression from baseline to 3 or 11 weeks after first ipilimumab dose, with a greater increase in the GI irAE group. Whereas CD177 is mostly an activation marker for neutrophils, CEACAM1 mediates adherence of activated neutrophils and other hematopoietic cells (NK and T cells) to cytokine-activated endothelium, [46,47] and has been suggested to play a role in immune-mediated diseases of the intestine. Elevated CEACAM1 expression has been reported in T cells of the lamina propria of small intestine in patients with celiac disease and in the large intestine of those with inflammatory bowel disease (IBD) [48].

The apparent association between neutrophil count, CD177 gene expression, and ipilimumab-associated GI irAEs led us to search for other neutrophil-associated genes in the microarray data. Degranulation is the process by which neutrophils release an assortment of proteins [49] such as MPO, DEFA4 [29] and ELANE [30] into the extracellular space. Most of these genes were included in a neutrophil module reported by Chaussabel et al. [33]. Although a trend for greater expression of these genes was found in those samples with the highest levels of CD177 expression (Figure 2A), there was no statistically significant linear correlation between their expression and that of CD177 (data not shown). In fact, the mean expression of most of these genes in week 3 samples was lower than baseline (Additional file 1: Table 2, left panel), when the greatest increase in CD177 was detected in some patients, suggesting that their expression might be repressed during the early neutrophil activation event. Conversely, a significant increase in the mean expression of these genes was observed at week 11 (Additional file 1: Table S1), supporting the notion that the degranulation process follows that of the neutrophil activation event. These observations were also confirmed in another data set from an independent ipilimumab clinical trial, CA184078, in which higher mean expression of CD177 and CEACAM1 were found in the GI irAE group.

In addition, our list of potential early predictors of GI irAEs shared a number of common elements with sets of genes reported to confer resistance to intravenous corticosteroid therapy in children with ulcerative colitis [50]. Genes shared between the two studies included CD177, CEACAM1, OLFM4, MMP8, BPI, CLC, HP, and LCN2. In that study, post-baseline samples were collected only three days after the start of the corticosteroid treatment with significant differential expression of these genes between the steroid-resistant and sensitive patients. In our study, the earliest post-baseline blood samples were collected 3 weeks after the first dose of ipilimumab, and the major changes in gene expression occurred within this time period, suggesting that it might be possible to detect this predictive profile at an even earlier time within this period. In any event, these changes tended to precede significant increases in the number of peripheral neutrophils, suggesting that the proliferation of neutrophils occurs after the activation event and that changes in gene expression may serve as more sensitive biomarkers than increase in peripheral blood absolute neutrophil count (PBANC).

Another interesting finding in our analysis was the considerable increase in the number and expression of immunoglobulin-related genes at 3 and 11 weeks after first dose of ipilimumab in patients who had GI irAEs. This is in accordance with previous reports on the inhibitory effects of CTLA-4 on immunoglobulin and cytokine production by plasma cells [51] or its inhibitory effect on CD4^+^ T cells that mediate T cell help to B cells during antibody production. CTLA-4 blockade by ipilimumab is likely to reduce this inhibition. In healthy people, the humoral response to enteric flora is maintained in homeostasis. Dysregulation of this homeostasis, manifested as increasing antibody levels to select enteric microorganisms, is characteristic of gastrointestinal disorders such as IBD but not acute GI inflammation (i.e., diverticulitis/infection) (7–9). In a previous report from the CA184007 trial, ipilimumab was found to induce antibody responses to selected enteric flora such as Pseudomonas anti-I2, *Saccharomyces cerevisiae*, and CBir flagellin. However, no strong association between a positive level of antibody responses toward these specific bacteria and GI irAEs was observed. Although gene expression profiling could not provide information regarding the specificity of the induced antibodies, it still indicates that in patients experiencing GI irAEs, the immunoglobulin production machinery had been turned on. In the absence of infections by external pathogens, this response may well be due to the generation of antibodies to self antigens or those expressed by the intestinal flora.

## Conclusions

We have identified early changes in gene expression in patients treated with ipilimumab that in some patients might predict the incidence of later GI irAEs. These gene expression changes, together with prior histopathologic examination of the affected tissue, point to an important role of neutrophils in the onset of GI irAEs in these patients. High expression of CD177 at week 3 was a very specific biomarker for grade 2+ GI irAEs, as all patients who had no such event displayed expression levels below a certain threshold (normalized expression level = 8). However, because of its low sensitivity as a biomarker, CD177 expression alone cannot be used to predict which patients will develop GI irAEs, which may occur in patients with low CD177 expression. The earliest on-treatment sample collection was 3 weeks after first ipilimumab dose. Therefore it is not clear how much increases in CD177 expression preceded the onset of GI irAEs. This study identified potential biomarkers of ipilimumab toxicity that have biological plausibility. However, validation in a larger controlled trial is needed to assess potential clinical utility.

## Abbreviations

CTLA-4: Cytotoxic T-lymphocyte antigen-4; irAEs: Immune-related adverse events; GI: Gastrointestinal; GI irAE: Gastrointestinal irAE; ANOVA: Analysis of variance; CD177: Cluster of differentiation 177; CEACAM1: Carcinoembryonic antigen-related cell adhesion molecule 1; FDA: Food and Drug Administration; APBNC: Absolute peripheral blood neutrophil counts; N: Number; RMA: Robust multi-array analysis; qPCR: Quantitative polymerase chain reaction; IPA: Ingenuity Pathway Analysis; CA: Clinical activity; OLFM4: Olfactomedin 4; ELANE: Elastase; CTSG: Cathepsin G; AZU1: Azurocidin 1; LTF: Lactoferrin [27]; CAMP: Cathelicidin; MPO: Myeloperoxidase; DEFA4: Defensin 4; BPI: Bactericidal/permeability increasing protein; IG: Immunoglobulin; NK: Natural killer; CXCL: Chemokine; TNF-α: Tumor necrosis factor-alpha; IBD: Inflammatory bowel disease; PEACAM-1: Platelet endothelial cell adhesion molecule-1

## Competing interests

Vafa Shahabi, David Berman, Scott D. Chasalow, Lisu Wang, Beihong Hu, Lisa Panting, Maria Jure-Kunkel, and Rui-Ru Ji are employees of Bristol-Myers Squibb, the manufacturer of ipilimumab. Zenta Tsuchihashi is a former employee of Bristol-Myers Squibb.

## Authors’ contributions

DB, ZT, SDC, MJK designed the two biomarker focused clinical trials. LW, BH, LP performed the PCR assays and prepared RNA for Affymetrix microarray analysis. MJK assisted in data analysis and interpretation. VF, SDC, ZT, RJ performed the data analysis and interpretation, and prepared the manuscript. All authors read and approved the final manuscript.

## Supplementary Material

Additional file 1 **Table 1.** Lists of potential predictive or early-predictive biomarkers. **(A)** Probe sets with ≥ 1.5-fold greater mean baseline expression in blood samples from patients with grade 2+ GI irAEs than in those from patients with no grade 2+ GI irAE (highlighted column). Mean expression ratio in the post-baseline samples as well as *P* values for the test of a difference at baseline in mean expression between the two GI irAE groups also are shown. **(B)** Probe sets with a mean expression ratio of at least 1.5 for the comparison of the GI irAE and No-GI irAE groups at week 3 (highlighted column). Mean expression ratio at other time points as well as *P* values for the test of a difference in week 3 mean expression between the two GI irAE groups also are shown. Mean expression ratio: positive values give (mean expression in the GI irAE group)/(mean expression in the No-GI irAE group); negative values give negative of (mean expression in the No-GI irAE group)/(mean expression in the GI irAE group). **Table 2.** Lists of potential pharmacodynamic biomarkers. Probe sets with ≥ 1.5-fold change in mean gene expression from baseline to week 3 (left panel) or week 11 (right panel; only the top 58 probe sets shown) in the GI irAE group (highlighted columns). Fold changes in the No-GI irAE group as well as *P* values for the test of a difference between baseline and post-baseline expression also are shown. Mean fold change: positive values give mean of (post-baseline expression)/(baseline expression); negative values give negative mean of (baseline expression)/(post-baseline expression). **Table 3.** Granule-associated gene expression profiles. **(A)** Mean expression ratio comparing the GI irAE and No-GI irAE groups for granule-associated genes at each time point. *P* value for the test of a difference in mean expression between the two GI irAE groups (averaged over the three time points) also is shown. **(B)** Mean fold change from baseline (BL) in the GI irAE and No-GI irAE groups for granule-associated genes. *P* value for the test of a difference in mean expression among the three time points (averaged over the two groups) is shown. For definitions of “mean expression ratio” and “mean fold change”, see legends for Tables 1 and 2, respectively. **Table S1.** Complete list of potential pharmacodynamic biomarkers. Probe sets with ≥ 1.5-fold mean change in gene expression from baseline to week 11 in the GI irAE group (highlighted column). Fold changes in the No-GI irAE group as well as *P* values for the test of a difference between baseline and post-baseline expression also are shown. Mean fold change: positive values give mean of (post-baseline expression)/(baseline expression); negative values give negative mean of (baseline expression)/(post-baseline expression). **Table S2.** Granule-associated gene expression profiles in CA184078. **(A)** Mean expression ratio comparing the GI irAE and No-GI irAE groups for granule-associated genes at each time point. *P* value for the test of a difference in mean expression between the two GI irAE groups (averaged over the three time points) also is shown. **(B)** Mean fold change from baseline (BL) in the GI irAE and No-GI irAE groups for granule-associated genes. *P* value for the test of a difference in mean expression among the three time points (averaged over the two groups) is shown. Mean expression ratio: positive values give (mean expression in the GI irAE group)/(mean expression in the No-GI irAE group); negative values give negative of (mean expression in the No-GI irAE group)/(mean expression in the GI irAE group). Mean fold change: positive values give mean of (post-baseline expression)/(baseline expression); negative values give negative mean of (baseline expression)/(post-baseline expression). Click here for file

Additional file 2: Figure S1 ROC curve of CD177 expression at week 3 as a predictor of GI irAE. The plot included 155 patients with known GI irAE status and CD177 expression values. Click here for file

## References

[B1] Korman AJ Peggs KS Allison JP Checkpoint blockade in cancer immunotherapy Adv Immunol 2006 90 297 339 16730267 10.1016/S0065-2776(06)90008-X PMC1951510

[B2] Hodi FS O’Day SJ McDermott DF Weber RW Sosman JA Haanen JB Gonzalez R Robert C Schadendorf D Hassel JC Akerley W Improved survival with ipilimumab in patients with metastatic melanoma N Engl J Med 2010 363 8 711 723 10.1056/NEJMoa1003466 20525992 PMC3549297

[B3] Weber J Overcoming immunologic tolerance to melanoma: targeting ctla-4 with ipilimumab (mdx-010) Oncologist 2008 13 Suppl 4 16 25 19001147 10.1634/theoncologist.13-S4-16

[B4] Beck KE Blansfield JA Tran KQ Feldman AL Hughes MS Royal RE Kammula US Topalian SL Sherry RM Kleiner D Quezado M Enterocolitis in patients with cancer after antibody blockade of cytotoxic t-lymphocyte-associated antigen 4 J Clin Oncol 2006 24 15 2283 2289 10.1200/JCO.2005.04.5716 16710025 PMC2140223

[B5] Berman D Parker SM Siegel J Chasalow SD Weber J Galbraith S Targan SR Wang HL Blockade of cytotoxic t-lymphocyte antigen-4 by ipilimumab results in dysregulation of gastrointestinal immunity in patients with advanced melanoma Cancer Immun 2010 10 11 21090563 PMC2999944

[B6] Weber J Thompson JA Hamid O Minor D Amin A Ron I Ridolfi R Assi H Maraveyas A Berman D Siegel J A randomized, double-blind, placebo-controlled, phase II study comparing the tolerability and efficacy of ipilimumab administered with or without prophylactic budesonide in patients with unresectable stage III or IV melanoma Clin Cancer Res 2009 15 17 5591 5598 10.1158/1078-0432.CCR-09-1024 19671877

[B7] Minor DR Chin K Kashani-Sabet M Infliximab in the treatment of anti-ctla4 antibody (ipilimumab) induced immune-related colitis Cancer Biother Radiopharm 2009 24 3 321 325 10.1089/cbr.2008.0607 19538054

[B8] Hamid O Schmidt H Nissan A Ridolfi L Aamdal S Hansson J Guida M Hyams DM Gomez H Bastholt L Chasalow SD A prospective phase II trial exploring the association between tumor microenvironment biomarkers and clinical activity of ipilimumab in advanced melanoma J Transl Med 2011 9 204 10.1186/1479-5876-9-204 22123319 PMC3239318

[B9] Weber J Hamid O Wolchok J Amin A Masson E Goldberg S Williams D Parker S Alaparthy S O’Day S Assessment of pharmacokinetic interaction between ipilimumab and chemotherapy in a randomized study35th ESMO Congress Abstract 1329P 2010

[B10] Ji RR Chasalow SD Wang L Hamid O Schmidt H Cogswell J Alaparthy S Berman D Jure-Kunkel M Siemers NO Jackson JR An immune-active tumor microenvironment favors clinical response to ipilimumab Cancer Immunol Immunother 2011 61 7 1019 1031 22146893 10.1007/s00262-011-1172-6 PMC11028506

[B11] Wang X Rickert M Garcia KC Structure of the quaternary complex of interleukin-2 with its alpha, beta, and gammac receptors Science 2005 310 5751 1159 1163 10.1126/science.1117893 16293754

[B12] Zhao X Lapalombella R Joshi T Cheney C Gowda A Hayden-Ledbetter MS Baum PR Lin TS Jarjoura D Lehman A Kussewitt D Targeting cd37-positive lymphoid malignancies with a novel engineered small modular immunopharmaceutical Blood 2007 110 7 2569 2577 10.1182/blood-2006-12-062927 17440052 PMC1988922

[B13] Kim SH Han SY Azam T Yoon DY Dinarello CA Interleukin-32: A cytokine and inducer of tnfalpha Immunity 2005 22 1 131 142 15664165 10.1016/j.immuni.2004.12.003

[B14] Zhang H Sun C Glogauer M Bokoch GM Human neutrophils coordinate chemotaxis by differential activation of rac1 and rac2 J Immunol 2009 183 4 2718 2728 10.4049/jimmunol.0900849 19625648 PMC3056163

[B15] McMahon LW Walsh CE Lambert MW Human alpha spectrin ii and the fanconi anemia proteins fanca and fancc interact to form a nuclear complex J Biol Chem 1999 274 46 32904 32908 10.1074/jbc.274.46.32904 10551855

[B16] Segura-Totten M Wilson KL Baf: Roles in chromatin, nuclear structure and retrovirus integration Trends Cell Biol 2004 14 5 261 266 10.1016/j.tcb.2004.03.004 15130582

[B17] Peelman LJ Chardon P Nunes M Renard C Geffrotin C Vaiman M Van Zeveren A Coppieters W van de Weghe A Bouquet Y The bat1 gene in the mhc encodes an evolutionarily conserved putative nuclear rna helicase of the dead family Genomics 1995 26 2 210 218 10.1016/0888-7543(95)80203-X 7601445

[B18] Gearhart MD Corcoran CM Wamstad JA Bardwell VJ Polycomb group and scf ubiquitin ligases are found in a novel bcor complex that is recruited to bcl6 targets Mol Cell Biol 2006 26 18 6880 6889 10.1128/MCB.00630-06 16943429 PMC1592854

[B19] Lee YS Chen CH Chao A Chen ES Wei ML Chen LK Yang KD Lin MC Wang YH Liu JW Eng HL Molecular signature of clinical severity in recovering patients with severe acute respiratory syndrome coronavirus (sars-cov) BMC Genomics 2005 10.1186/1471-2164-6-132 PMC1262710 16174304

[B20] Fujibuchi T Abe Y Takeuchi T Imai Y Kamei Y Murase R Ueda N Shigemoto K Yamamoto H Kito K Aip1/wdr1 supports mitotic cell rounding Biochem Biophys Res Commun 2005 327 1 268 275 10.1016/j.bbrc.2004.11.156 15629458

[B21] Tebar F Bohlander SK Sorkin A Clathrin assembly lymphoid myeloid leukemia (calm) protein: Localization in endocytic-coated pits, interactions with clathrin, and the impact of overexpression on clathrin-mediated traffic Mol Biol Cell 1999 10 8 2687 2702 10436022 10.1091/mbc.10.8.2687 PMC25500

[B22] Bernstein AM Whiteheart SW Identification of a cellubrevin/vesicle associated membrane protein 3 homologue in human platelets Blood 1999 93 2 571 579 9885218

[B23] Lalezari P Murphy GB Allen FH Jr Nb1, a new neutrophil-specific antigen involved in the pathogenesis of neonatal neutropenia J Clin Invest 1971 50 5 1108 1115 10.1172/JCI106582 5552408 PMC292033

[B24] Stroncek DF Shankar RA Noren PA Herr GP Clement LT Analysis of the expression of nb1 antigen using two monoclonal antibodies Transfusion 1996 36 2 168 174 10.1046/j.1537-2995.1996.36296181931.x 8614969

[B25] Stroncek DF Skubitz KM McCullough JJ Biochemical characterization of the neutrophil-specific antigen nb1 Blood 1990 75 3 744 755 2153425

[B26] Rosenbauer F Wagner K Zhang P Knobeloch KP Iwama A Tenen DG Pdp4, a novel glycoprotein secreted by mature granulocytes, is regulated by transcription factor pu.1 Blood 2004 103 11 4294 4301 10.1182/blood-2003-08-2688 14962908

[B27] Miyauchi J Watanabe Y Immunocytochemical localization of lactoferrin in human neutrophils. An ultrastructural and morphometrical study Cell Tissue Res 1987 247 2 249 258 3028636 10.1007/BF00218306

[B28] Falloon J Gallin JI Neutrophil granules in health and disease J Allergy Clin Immunol 1986 77 5 653 662 10.1016/0091-6749(86)90404-5 3009589

[B29] Gabay JE Scott RW Campanelli D Griffith J Wilde C Marra MN Seeger M Nathan CF Antibiotic proteins of human polymorphonuclear leukocytes Proc Natl Acad Sci U S A 1989 86 14 5610 5614 10.1073/pnas.86.14.5610 2501794 PMC297672

[B30] Korkmaz B Horwitz MS Jenne DE Gauthier F Neutrophil elastase, proteinase 3, and cathepsin g as therapeutic targets in human diseases Pharmacol Rev 2010 62 4 726 759 10.1124/pr.110.002733 21079042 PMC2993259

[B31] Zhao L Furebring M Xu S Venge P Subcellular localization and mobilization of carcinoembryonic antigen-related cell adhesion molecule 8 in human neutrophils Br J Haematol 2004 125 5 666 673 10.1111/j.1365-2141.2004.04963.x 15147383

[B32] Skubitz KM Skubitz AP Interdependency of ceacam-1, -3, -6, and −8 induced human neutrophil adhesion to endothelial cells J Transl Med 2008 6 78 10.1186/1479-5876-6-78 19077207 PMC2628881

[B33] Chaussabel D Quinn C Shen J Patel P Glaser C Baldwin N Stichweh D Blankenship D Li L Munagala I Bennett L A modular analysis framework for blood genomics studies: application to systemic lupus erythematosus Immunity 2008 29 1 150 164 10.1016/j.immuni.2008.05.012 18631455 PMC2727981

[B34] Robert C Thomas L Bondarenko I O’Day S DJ M Garbe C Lebbe C Baurain JF Testori A Grob JJ Davidson N Ipilimumab plus dacarbazine for previously untreated metastatic melanoma N Engl J Med 2011 364 26 2517 2526 10.1056/NEJMoa1104621 21639810

[B35] Shioya M Nishida A Yagi Y Ogawa A Tsujikawa T Kim-Mitsuyama S Takayanagi A Shimizu N Fujiyama Y Andoh A Epithelial overexpression of interleukin-32alpha in inflammatory bowel disease Clin Exp Immunol 2007 149 3 480 486 10.1111/j.1365-2249.2007.03439.x 17590175 PMC2219317

[B36] Cagnard N Letourneur F Essabbani A Devauchelle V Mistou S Rapinat A Decraene C Fournier C Chiocchia G Interleukin-32, ccl2, pf4f1 and gfd10 are the only cytokine/chemokine genes differentially expressed by in vitro cultured rheumatoid and osteoarthritis fibroblast-like synoviocytes Eur Cytokine Netw 2005 16 4 289 292 16464743

[B37] Monsurro V Wang E Yamano Y Migueles SA Panelli MC Smith K Nagorsen D Connors M Jacobson S Marincola FM Quiescent phenotype of tumor-specific cd8+ t cells following immunization Blood 2004 104 7 1970 1978 10.1182/blood-2004-02-0525 15187028

[B38] Jaeschke H Smith CW Clemens MG Ganey PE Roth RA Mechanisms of inflammatory liver injury: adhesion molecules and cytotoxicity of neutrophils Toxicol Appl Pharmacol 1996 139 2 213 226 10.1006/taap.1996.0160 8806837

[B39] Segura RM Alegre J Varela E Marti R Surinach JM Jufresa J Armadans L Pascual C Fernandez de Sevilla T Interleukin-8 and markers of neutrophil degranulation in pleural effusions Am J Respir Crit Care Med 1998 157 5 Pt 1 1565 1572 9603139 10.1164/ajrccm.157.5.9711116

[B40] Jacobs L Nawrot TS de Geus B Meeusen R Degraeuwe B Bernard A Sughis M Nemery B Panis LI Subclinical responses in healthy cyclists briefly exposed to traffic-related air pollution: an intervention study Environ Health 2010 Published online 2010 October 25 10.1186/1476-069X-9-64 PMC2984475 20973949

[B41] De Larco JE Wuertz BR Furcht LT The potential role of neutrophils in promoting the metastatic phenotype of tumors releasing interleukin-8 Clin Cancer Res 2004 10 15 4895 4900 10.1158/1078-0432.CCR-03-0760 15297389

[B42] Waugh DJ Wilson C The interleukin-8 pathway in cancer Clin Cancer Res 2008 14 21 6735 6741 10.1158/1078-0432.CCR-07-4843 18980965

[B43] Follin P Wymann MP Dewald B Ceska M Dahlgren C Human neutrophil migration into skin chambers is associated with production of nap-1/il8 and c5a Eur J Haematol 1991 47 1 71 76 1868917 10.1111/j.1600-0609.1991.tb00564.x

[B44] Gohring K Wolff J Doppl W Schmidt KL Fenchel K Pralle H Sibelius U Bux J Neutrophil cd177 (nb1 gp, hna-2a) expression is increased in severe bacterial infections and polycythaemia vera Br J Haematol 2004 126 2 252 254 10.1111/j.1365-2141.2004.05027.x 15238147

[B45] Sachs UJ Andrei-Selmer CL Maniar A Weiss T Paddock C Orlova VV Choi EY Newman PJ Preissner KT Chavakis T Santoso S The neutrophil-specific antigen cd177 is a counter-receptor for platelet endothelial cell adhesion molecule-1 (cd31) J Biol Chem 2007 282 32 23603 23612 10.1074/jbc.M701120200 17580308

[B46] Gray-Owen SD Blumberg RS Ceacam1: contact-dependent control of immunity Nature Rev 2006 6 6 433 446 10.1038/nri1864 16724098

[B47] Skubitz KM Skubitz AP Two new synthetic peptides from the n-domain of ceacam1 (cd66a) stimulate neutrophil adhesion to endothelial cells Biopolymers 2011 96 1 25 31 10.1002/bip.21447 20560140

[B48] Costello CM Mah N Hasler R Rosenstiel P Waetzig GH Hahn A Lu T Gurbuz Y Nikolaus S Albrecht M Hampe J Dissection of the inflammatory bowel disease transcriptome using genome-wide cDNA microarrays PLoS Med 2005 2 8 e199 10.1371/journal.pmed.0020199 16107186 PMC1188246

[B49] Witko-Sarsat V Rieu P Descamps-Latscha B Lesavre P Halbwachs-Mecarelli L Neutrophils: molecules, functions and pathophysiological aspects Lab Invest 2000 80 5 617 653 10.1038/labinvest.3780067 10830774

[B50] Kabakchiev B Turner D Hyams J Mack D Leleiko N Crandall W Markowitz J Otley AR Xu W Hu P Griffiths AM Gene expression changes associated with resistance to intravenous corticosteroid therapy in children with severe ulcerative colitis PLoS One 2010 5 9 e13085 10.1371/journal.pone.0013085 20941359 PMC2948001

[B51] Merlo A Tenca C Fais F Battini L Ciccone E Grossi CE Saverino D Inhibitory receptors cd85j, lair-1, and cd152 down-regulate immunoglobulin and cytokine production by human b lymphocytes Clin Diagn Lab Immunol 2005 12 6 705 712 15939744 10.1128/CDLI.12.6.705-712.2005 PMC1151979

