# Supplementary material for: Gene expression profiling of whole blood in ipilimumab-treated patients for identification of potential biomarkers of immune-related gastrointestinal adverse events
Source: J Transl Med. 2013 Mar 22;11:75. doi: 10.1186/1479-5876-11-75 (PMC3637501; doi:10.1186/1479-5876-11-75)
Supplement: Additional file 2: Figure S1 — ROC curve of CD177 expression at week 3 as a predictor of GI irAE. The plot included 155 patients with known GI irAE status and CD177 expression values. [file 1479-5876-11-75-S2.pptx]

## Slide 1
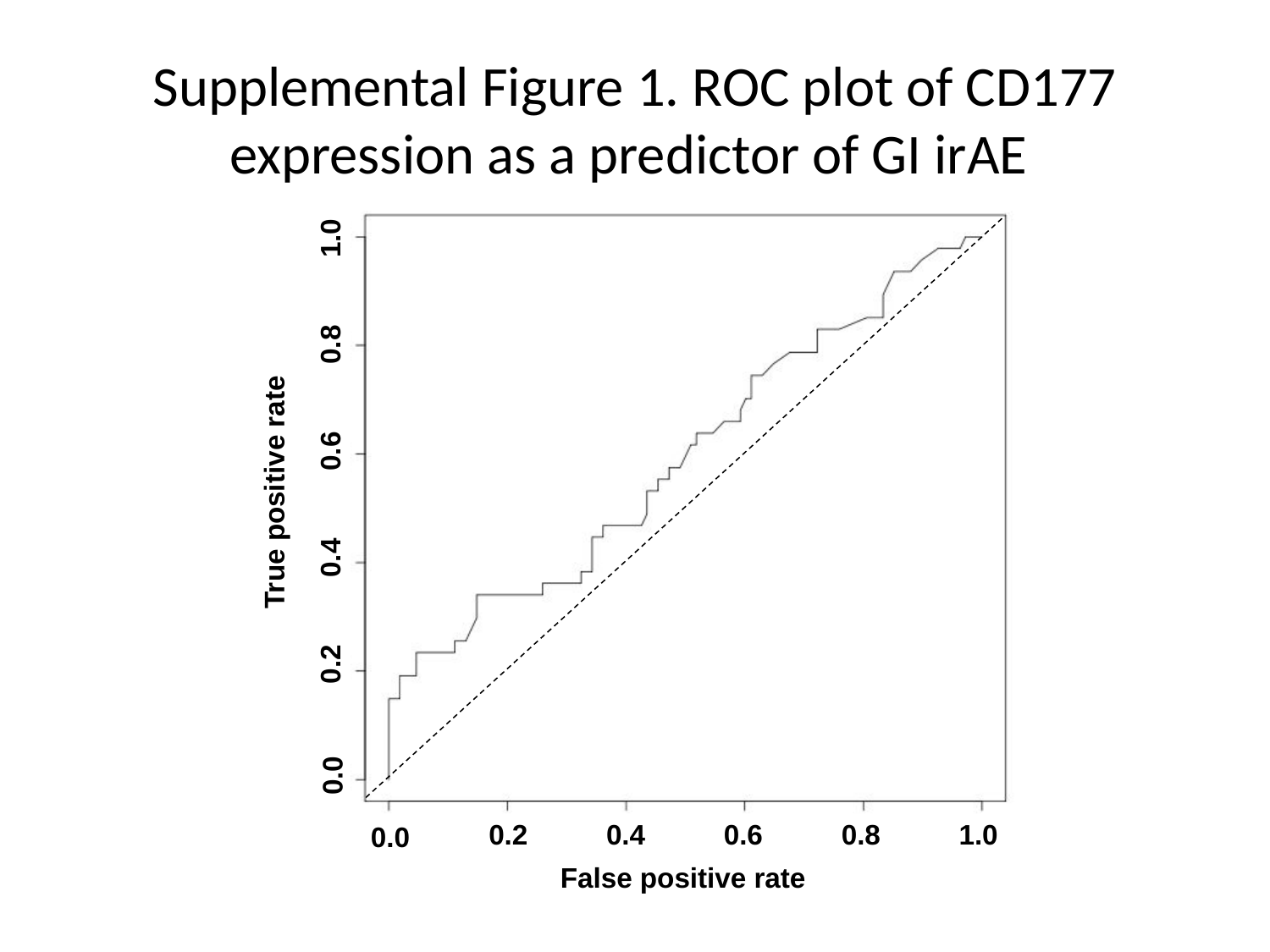

# Supplemental Figure 1. ROC plot of CD177 expression as a predictor of GI irAE
1.0
0.8
0.6
0.4
0.2
0.0
True positive rate
0.2
0.4
0.6
0.8
1.0
0.0
False positive rate
